# Supplementary material for: GAS41 modulates ferroptosis by anchoring NRF2 on chromatin
Source: Nat Commun. 2024 Mar 21;15:2531. doi: 10.1038/s41467-024-46857-w (PMC10957913; doi:10.1038/s41467-024-46857-w)
Supplement: Supplementary file 1 — Supplementary Information [file 41467_2024_46857_MOESM1_ESM.pdf]

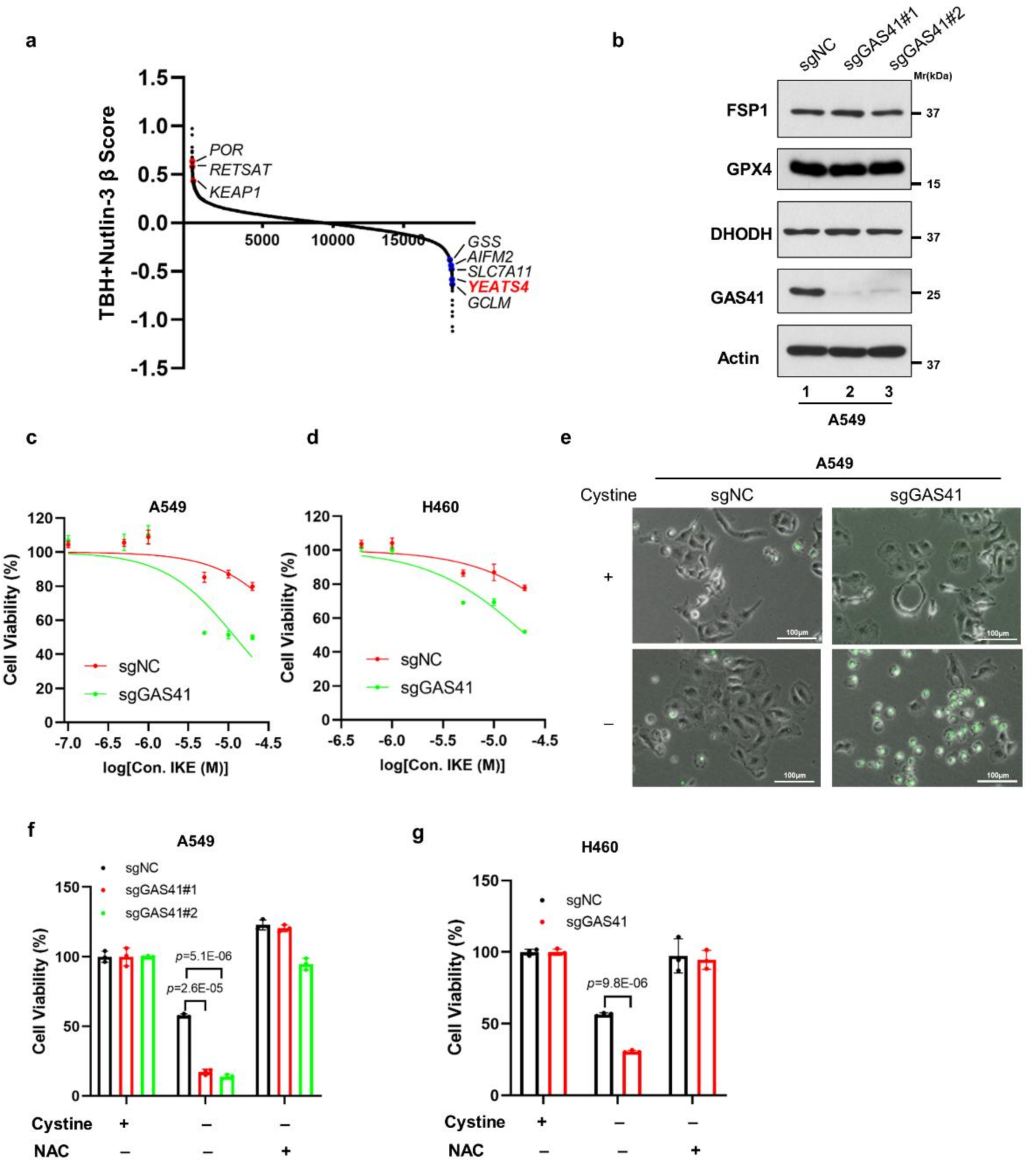

Supplementary Fig.1: Identification GAS41 as a ferroptosis repressor upon ROS stress

a Plot showing the β scores (TBH vs. T_{14}) for all targeted genes by human genome wide CRISPR knockout library. Red dots represent known significantly positive selected genes. Blue dots represent known significantly negative selected genes. *YEATS4* is highlighted in red.

b Western blot of FSP1, GPX4, and DHODH protein levels in A549 cells sgNC and sgGAS41 cells.

c Cell viability of sgNC and sgGAS41 A549 cells treated with IKE for 24h.

d Cell viability of sgNC and sgGAS41 H460 cells treated with IKE for 24h.

e Representative phase-contrast SYTOX stained images of sgNC and sgGAS41 A549 cells incubated with or without cystine for 48h. Scale bars, 100 μm.

f Cell viability of sgNC and sgGAS41 A549 cells starved with cystine for 48h.

g Cell viability of sgNC and sgGAS41 H460 cells starved with cystine for 36h.

Data are mean ± SD of n = 3 independent biological repeats. *p* values were calculated using unpaired, two-tailed Student's *t*-test. The experiments above (**b**, **e**) were repeated three times with similar results and representative images are shown. Source data are provided as a Source Data file.

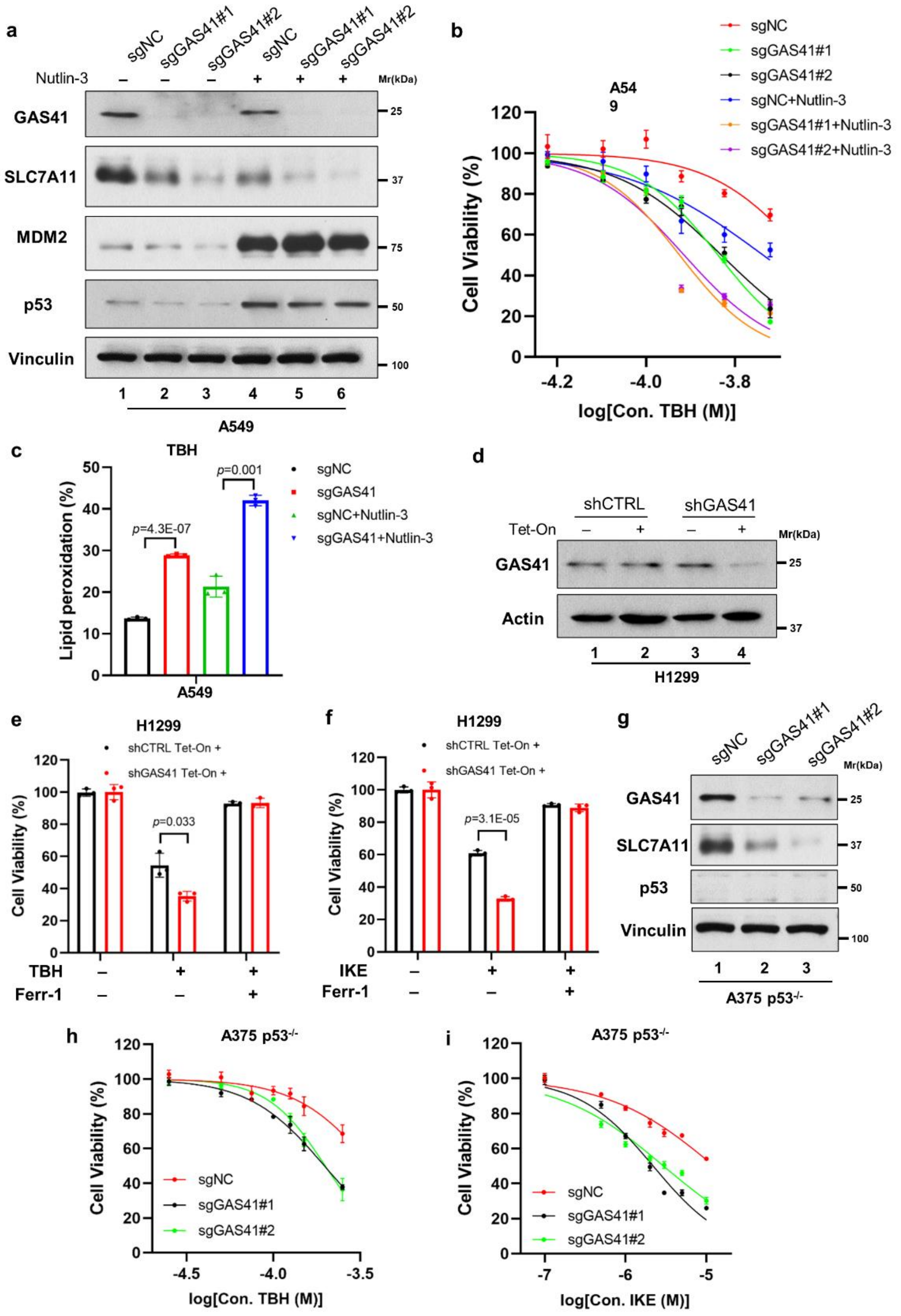

Supplementary Fig. 2: GAS41 suppresses ferroptosis in a p53-independent manner

a Western blot of GAS41 and SLC7A11 protein levels in sgNC and sgGAS41 A549 cells with or without Nutlin-3 (5 μM) treatment for 24h.

b Cell viability of sgNC and sgGAS41 A549 cells pre-incubated with Nutlin-3 (5 μM) for 12h then treated with TBH for 6h.

c Assessment of lipid peroxidation by flow cytometry after C11-BODIPY staining

in sgNC and sgGAS41 A549 cells pre-incubated Nutlin-3 for 12h then treated with TBH (120 μM) for 4h.

d Western blot of GAS41 protein levels in H1299 shControl (shCTRL) and shGAS41 Tet-On inducible cells without or with doxycycline (0.2 μg/mL) as indicated for 72 h.

e Cell viability of H1299 shCTRL and shGAS41 Tet-On inducible cells pre-incubated with doxycycline (0.2 μg/mL) for 72h then treated with TBH (100 μM) for 6 h.

f Cell viability of H1299 shCTRL and shGAS41 Tet-On inducible cells pre-incubated with doxycycline (0.2 μg/mL) for 72h then treated with IKE (4 μM) for 12 h.

g Western blot of GAS41 protein levels of A375 $p53^{-/-}$ sgNC and sgGAS41 cells generated using control sgRNA and two individual targeting GAS41 sgRNA, respectively.

h Cell viability of A375 $p53^{-/-}$ sgNC and sgGAS41 cells treated with TBH for 6h.

i Cell viability of A375 $p53^{-/-}$ sgNC and sgGAS41 cells treated with IKE for 24h.

Data are mean ± SD of n = 3 independent biological repeats. *p* values were calculated using unpaired, two-tailed Student's *t* test. Western blot experiments above (**a**, **d**, and **g**) were repeated three times with similar results and representative images are shown. Source data are provided as a Source Data file.

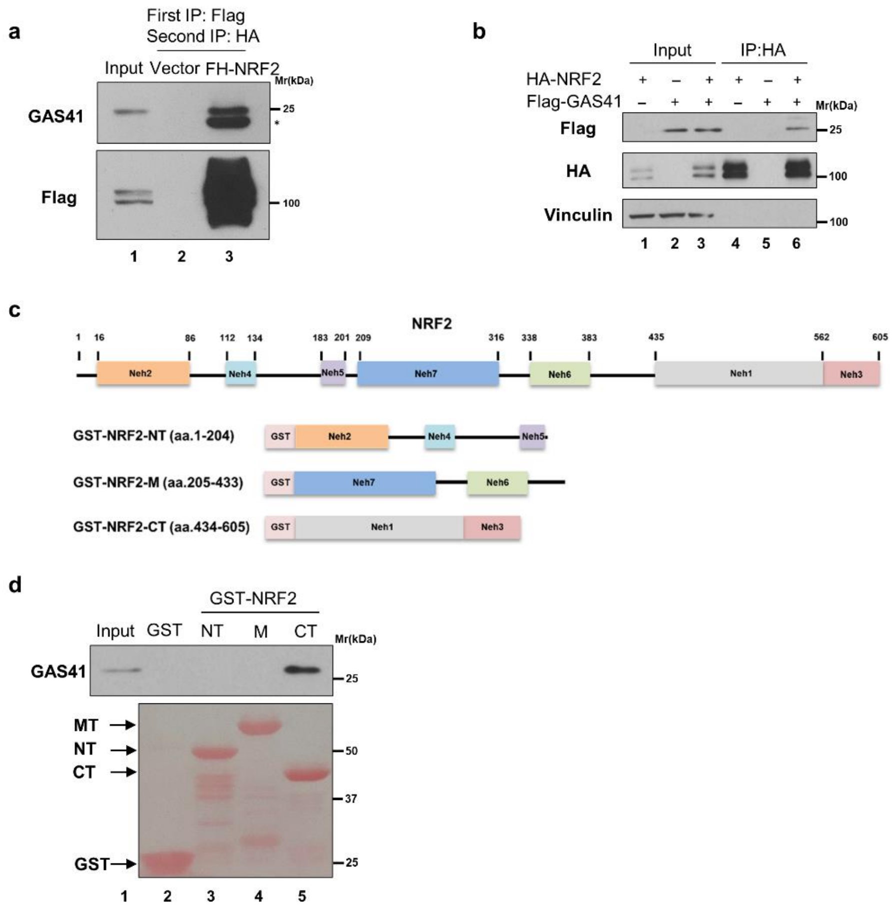

Supplementary Fig.3: GAS41 interacts NRF2 *in vivo* and *in vitro*

a Western blot of GAS41 in purified NRF2-associated protein by two steps immunoprecipitations: first step IP by Flag and second step IP by HA. * : non-specific band.

b Western blot of the interaction between overexpressed Flag-GAS41 with HA-NRF2 in HEK293T cells.

c Schematic diagram of the NRF2 domains and NRF2 fragments used in this study. GST-NRF2-NT contains NRF2-ECH homology (Neh) domain 2, 4, and 5, AA. 1-204; GST-NRF2-MT contains Neh domain 7 and 6, AA. 205-433; GST-NRF2-CT contains Neh domain 1 and 3, AA. 434-605.

d *In vitro* binding assay of purified Flag-GAS41 and GST-NRF2 fragments shown in (**c**).

Western blot experiments above (**a**, **b**, and **d**) were repeated three times with similar results and representative images are shown. Source data are provided as a Source Data file.

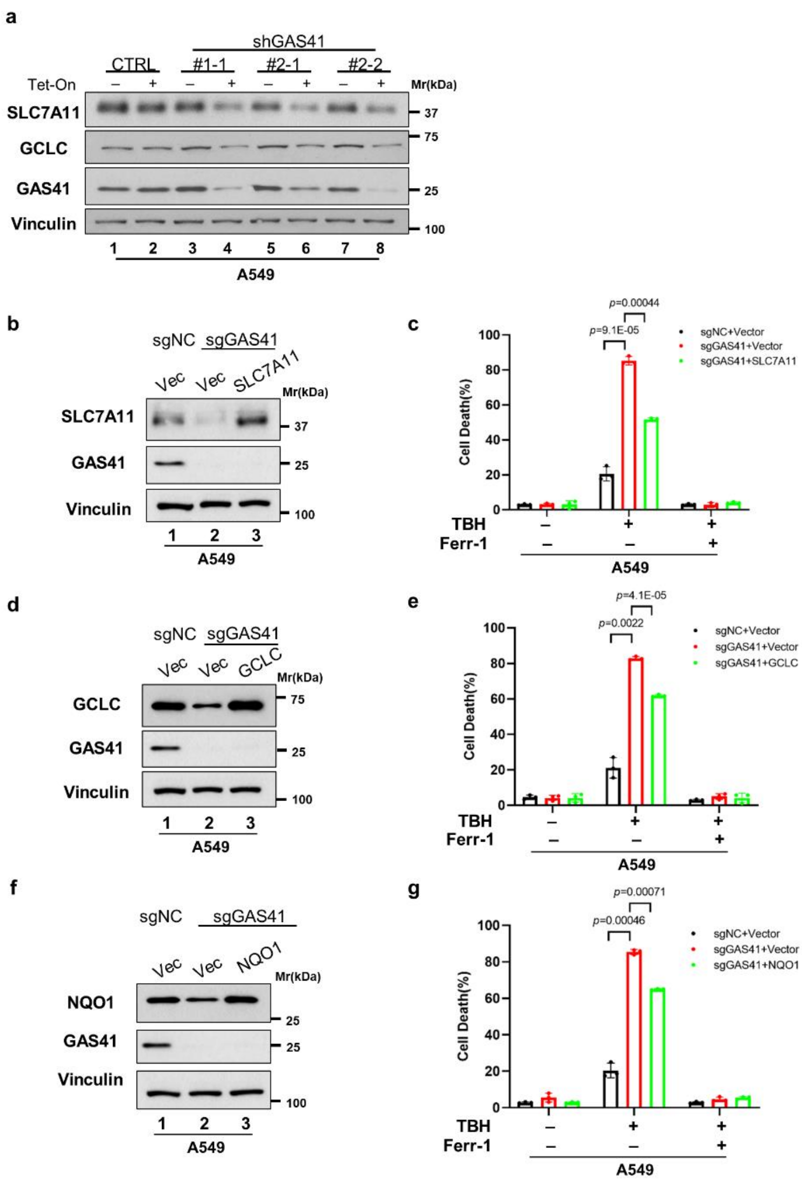

Supplementary Fig.4: GAS41 promotes NRF2 transcriptional ability on antioxidant genes

a Western blot of SLC7A11, GCLC, and GAS41 protein levels in A549 shCTRL and shGAS41 Tet-On inducible cells incubated without or with doxycycline (0.2 μg/mL) for 72h.

b Western blot of SLC7A11 protein levels of A549 sgNC re-expressed with vector, sgGAS41 cells re-expressed with vector or SLC7A11 expression plasmid.

c Cell death of A549 sgNC re-expressed with vector, sgGAS41 cells re-expressed with vector or SLC7A11 expression plasmid under TBH treatment (120 μM) for 6h.

d Western blot of GCLC protein levels of A549 sgNC re-expressed with vector, sgGAS41 cells re-expressed with vector or GCLC expression plasmid.

e Cell death of A549 sgNC re-expressed with vector, sgGAS41 cells re-expressed with vector or GCLC expression plasmid under TBH treatment (120

μM) for 6h.

f Western blot of NQO1 protein levels of A549 sgNC re-expressed with vector, sgGAS41 cells re-expressed with vector or NQO1 expression plasmid.

g Cell death of A549 sgNC re-expressed with vector, sgGAS41 cells re-expressed with vector or NQO1 expression plasmid under TBH treatment (120 μM) for 6h.

Data are mean ± SD of n = 3 independent biological repeats. *p* values were calculated using unpaired, two-tailed Student's *t* test. Western blot experiments above (**a**, **b**, **d**, and **f**) were repeated three times with similar results and representative images are shown. Source data are provided as a Source Data file.

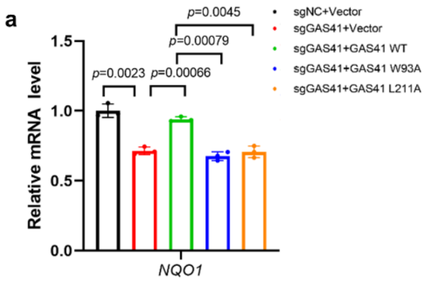

Supplementary Fig.5: The interaction between GAS41 and NRF2 is required for NRF2 transcriptional ability

a RT-qPCR analysis of *NQO1* mRNA levels of A549 sgNC re-expressed with vector, sgGAS41 cells re-expressed with vector, GAS41 WT, GAS41 W93A mutant or GAS41 L211A mutant.

Data are mean ± SD of n = 3 independent biological repeats. *p* values were calculated using unpaired, two-tailed Student's *t*-test. Source data are provided as a Source Data file.

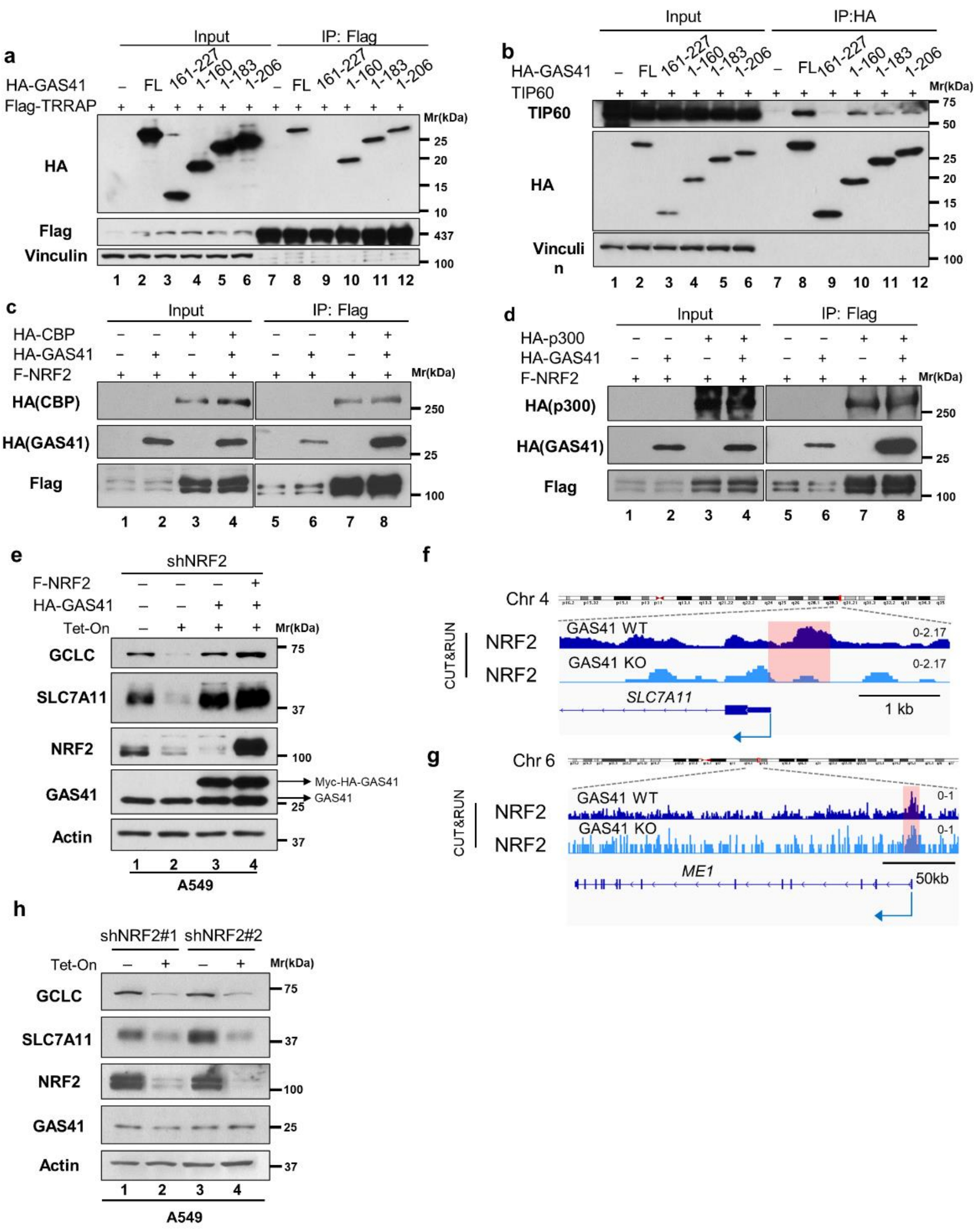

Supplementary Fig.6: The regulatory interplay of NRF2 and GAS41 in transcriptional regulation

a Western blot analysis of interaction between TRRAP and GAS41 fragments (as shown in **Fig. 2g**) in HEK293T cells. FL, full length, amino acids from 1 to 227.

b Western blot analysis of interaction between TIP60 and GAS41 fragments (as shown in **Fig. 2g**) in HEK293T cells. FL, full length, amino acids from 1 to 227.

c Western blot of interaction between overexpressed Flag-NRF2 with HA-CBP in presence or absence of HA-GAS41 from HEK293T cells.

d Western blot of interaction between overexpressed Flag-NRF2 with HA-p300 in presence or absence of HA-GAS41 from HEK293T cells.

e Western blot of overexpressed HA-GAS41 and Flag-NRF2 in A549 shNRF2 Tet-on inducible cells pre-incubated without or with doxycycline (0.2 μg/mL) for

48h, then transfected with HA tagged GAS41 expressing plasmid along with vector or Flag tagged NRF2 expressing plasmid.

f,**g** Snapshot of NRF2 CUT&RUN signal in A549 sgNC and sgGAS41 cells at *SLC7A11* (**f**) and *ME1* (**g**) genes loci.

h Western blot of GAS41, NRF2, SLC7A11, and GCLC protein levels in shNRF2 A549 cells incubated with or without doxycycline (0.2 μg/mL) for 72h.

Western blot experiments above (**a**-**e** and **h**) were repeated three times with similar results and representative images are shown. Source data are provided as a Source Data file.

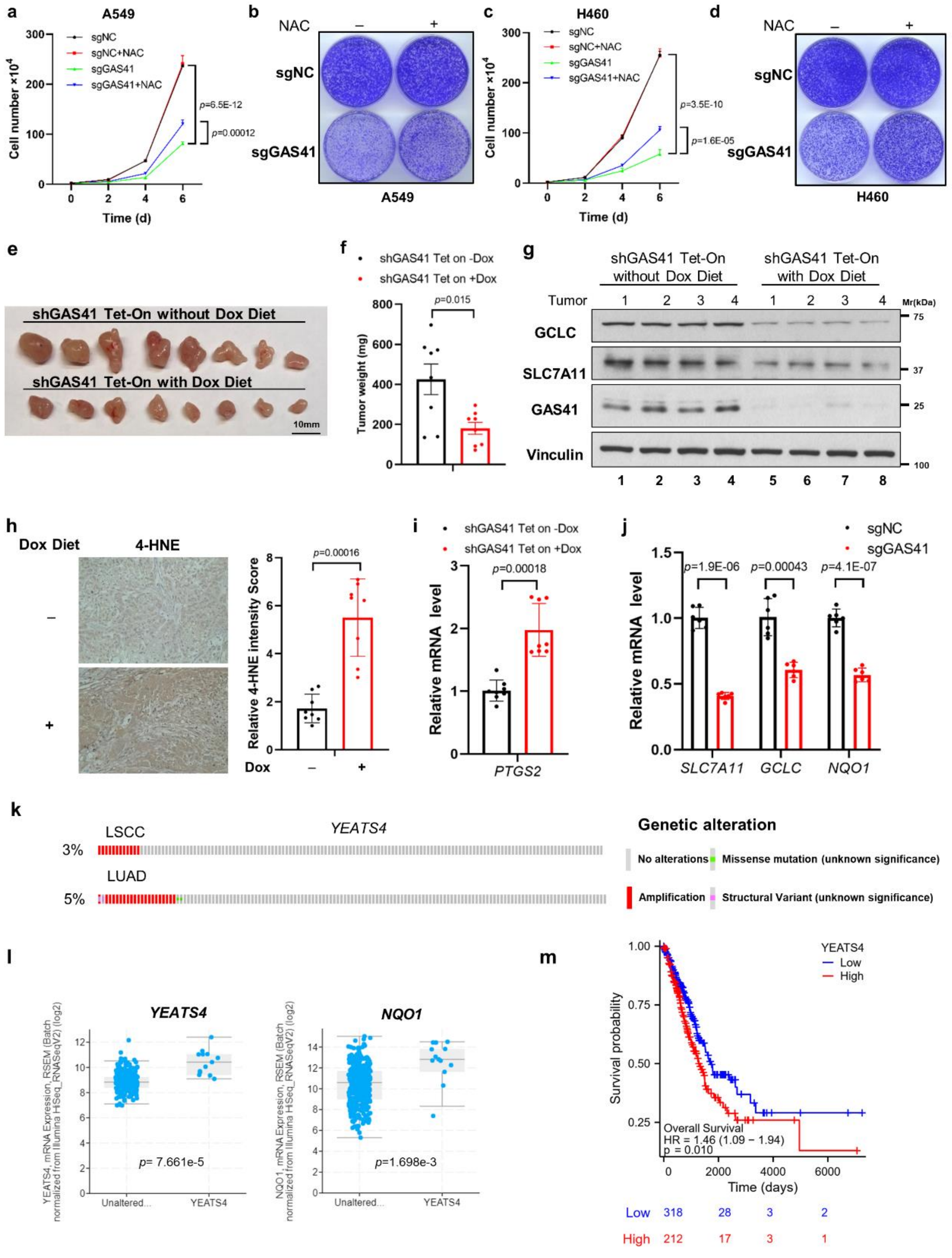

Supplementary Fig.7: Loss of GAS41 promotes tumor suppression, at least partially through ferroptosis *in vivo*

a Cell proliferation rate of sgNC and sgGAS41 A549 cells incubated with or without NAC supplement (500 μM).

b Representative colony formation images of sgNC and sgGAS41 A549 cells incubated with or without NAC supplement (500 μM) for 10 days.

c Cell proliferation rate of sgNC and sgGAS41 H460 cells incubated with or without NAC supplement (500 μM).

d Representative colony formation images of sgNC and sgGAS41 H460 cells incubated with or without NAC supplement (500 μM) for 10 days.
e Image of xenograft tumors from A549 shGAS41 Tet-On inducible cells.
f Quantification of tumor weight from the experiments in (**e**).
g Western blot of GCLC, SLC7A11, and GAS41 protein levels of tumor tissues in (**e**).
h Representative staining image (left panel) and quantification (right panel) of 4-HNE intensity from tumor tissues in (**e**).
i RT-qPCR analysis of *PTGS2* mRNA levels from tumor tissues in (**e**).
j RT-qPCR analysis of *GCLC*, *SLC7A11*, and *NQO1* mRNA levels from tumor tissues described in (**Fig. 6a**).
k Mutation type of *YEATS4* from TCGA-LSCC (n=469) and TCGA-LUAD (n=507).
l Box plot of *YEATS4* and *NQO1* mRNA expression of *YEATS4*-unaltered (*NFE2L2*, *KEAP1, YEATS4* wildtype, n = 328) or -amplified (n = 13) patients from TCGA-LSCC (lung squamous cell carcinoma) analyzed by cBioPortal. The median value is shown in the box. The whiskers indicate the value of minima and maxima, and the box bounds indicate the value of the first quartile or third quartile.
m Kaplan–Meier plots of TCGA-LUAD patients stratified by *YEATS4* expression levels.
For (**a**, **c**) data are mean ± SD of n = 5, for (**h** right panel, **i**) data are mean ± SD of n = 8, and for **j**, data are mean ± SD of n = 6 independent biological repeats. For **f**, data are mean ± SEM of n = 8 independent tumor samples. *p* values were calculated using unpaired, two-tailed Student's *t*-test. Source data are provided as a Source Data file.

Supplementary Table 1. The primers used for qPCR

**Primer Description**	**Sequence (5'-3')**
GCLC-Forward primer in gene expression	GAGGTCAAACCCAACCCAGT
GCLC-Reverse primer in gene expression	AAGGTACTGAAGCGAGGGTG
NQO1-Forward primer in gene expression	CAAAGGACCCTTCCGGAGTAA
NQO1-Reverse primer in gene expression	ACTTGGAAGCCACAGAAATGC
YEATS4-Forward primer in gene expression	CTAACATTAGGAGCCTAT
YEATS4-Reverse primer in gene expression	TATGTCTTTTGCTTGGTC
SLC7A11-Forward primer in gene expression	TCATTGGAGCAGGAATCTTCA
SLC7A11-Reverse primer in gene expression	TTCAGCATAAGACAAAGCTCCA
TIP60-Forward primer in gene expression	AATGTGGCCTGCATCCTAAC
TIP60-Reverse primer in gene expression	TGTTTTCCCTTCCACTTTGG
TRRAP-Forward primer in gene expression	ATGATGCAAGAAGTTAGTGAAAA
TRRAP-Reverse primer in gene expression	GAAGATGTTCGTTGGTTGGTA
PTGS2-Forward primer in gene expression	CTTCACGCATCAGTTTTTCAAG
PTGS2-Reverse primer in gene expression	TCACCGTAAATATGATTTAAGTCCAC
ACTB-Forward primer in gene expression	TCCATCATGAAGTGTGACG
ACTB-Reverse primer in gene expression	TACTCCTGCTTGCTGATCCAC
GCLC-Forward primer in ChIP-qPCR	GGACTGAGACTTTGCCCTAA
GCLC-Reverse primer in ChIP-qPCR	CAGTTGTTGTGATACAGCCC
SLC7A11-Forward primer in ChIP-qPCR	GTGGCTGATGCAAACCTG
SLC7A11-Reverse primer in ChIP-qPCR	CCAGCTCAGCTTCCTCAT
