# Supplementary material for: GAS41 modulates ferroptosis by anchoring NRF2 on chromatin
Source: Nat Commun. 2024 Mar 21;15:2531. doi: 10.1038/s41467-024-46857-w (PMC10957913; doi:10.1038/s41467-024-46857-w)
Supplement: Supplementary file 3 — Reporting Summary [file 41467_2024_46857_MOESM3_ESM.pdf]

nature portfolio

Corresponding author(s): WEI GU

Last updated by author(s): Mar 1, 2024

Reporting Summary

Nature Portfolio wishes to improve the reproducibility of the work that we publish. This form provides structure for consistency and transparency in reporting. For further information on Nature Portfolio policies, see our Editorial Policies and the Editorial Policy Checklist.

Statistics

For all statistical analyses, confirm that the following items are present in the figure legend, table legend, main text, or Methods section.

n/a	Confirmed	
☐	☒	The exact sample size (*n*) for each experimental group/condition, given as a discrete number and unit of measurement
☐	☒	A statement on whether measurements were taken from distinct samples or whether the same sample was measured repeatedly
☐	☒	The statistical test(s) used AND whether they are one- or two-sided<br>*Only common tests should be described solely by name; describe more complex techniques in the Methods section.*
☒	☐	A description of all covariates tested
☒	☐	A description of any assumptions or corrections, such as tests of normality and adjustment for multiple comparisons
☐	☒	A full description of the statistical parameters including central tendency (e.g. means) or other basic estimates (e.g. regression coefficient) AND variation (e.g. standard deviation) or associated estimates of uncertainty (e.g. confidence intervals)
☐	☒	For null hypothesis testing, the test statistic (e.g. *F*, *t*, *r*) with confidence intervals, effect sizes, degrees of freedom and *P* value noted<br>*Give P values as exact values whenever suitable.*
☒	☐	For Bayesian analysis, information on the choice of priors and Markov chain Monte Carlo settings
☒	☐	For hierarchical and complex designs, identification of the appropriate level for tests and full reporting of outcomes
☒	☐	Estimates of effect sizes (e.g. Cohen's *d*, Pearson's *r*), indicating how they were calculated

Our web collection on statistics for biologists contains articles on many of the points above.

Software and code

Policy information about availability of computer code

Data collection	Deep sequencing for CRISPR-Cas9 screen or CUT&RUN was collected from Illumina HiSeq2500 or Illumina NextSeq 500 platforms by Columbia University Genome Center.<br>Applied Biosystems 7500 Fast Dx Real-Time PCR Instrument was used for qPCR analysis.<br>Attune NxT Acoustic Focusing Cytometer (Thermo Fisher Scientific) was used for lipid peroxidation analysis.<br>IHC pictures were collected by Nikon ECLIPSE Ni Microscope.<br>GloMax Discover Microplate Reader (Promega) was used for analysis of cellular ATP levels, GSH production and Dual-luciferase Assay.<br>Olympus IX51Microscope was used for cell death analysis.
Data analysis	MAGeCK (version 0.5.7) software was used for CRISPR Cas9 screen analysis. FACS data was analyzed by FlowJo v10. Density of immunohistochemistry images was analyzed by Image-Pro Plus software. Plots and statistical analysis was determined by Graphpad Prism 8.0.2 or Microsoft Excel. Gene tracks were visualized with IGV (version 2.17.0). Trim Galore (version 0.6.7), Bowtie2 (version 2.2.4), BEDTools (version 2.29.2), bedGraphToBigWig (version 359), and SEACR2 (version 1.3) were used for CUT&RUN analysis.

For manuscripts utilizing custom algorithms or software that are central to the research but not yet described in published literature, software must be made available to editors and reviewers. We strongly encourage code deposition in a community repository (e.g. GitHub). See the Nature Portfolio guidelines for submitting code & software for further information.

Data

Policy information about availability of data

All manuscripts must include a data availability statement. This statement should provide the following information, where applicable:

- Accession codes, unique identifiers, or web links for publicly available datasets
- A description of any restrictions on data availability
- For clinical datasets or third party data, please ensure that the statement adheres to our policy

The CUT&RUN data generated in this study have been deposited in the GEO database under accession code GSE256462 (https://www.ncbi.nlm.nih.gov/geo/query/acc.cgi?acc=GSE256462). All other data needed to evaluate the conclusions in this study are available in the study and its Supplementary Information. The following public databases were used in this study (see Methods for more details): cBioPortal for Cancer Genomics (https://www.cbioportal.org/) and The Cancer Genome Atlas Program (TCGA) (https://www.cancer.gov/ccg/research/genome-sequencing/tcga). Source data are provided with this paper in the Source Data files. All other data and materials are available from the corresponding author upon reasonable request.

Research involving human participants, their data, or biological material

Policy information about studies with human participants or human data. See also policy information about sex, gender (identity/presentation), and sexual orientation and race, ethnicity and racism.

Reporting on sex and gender	No human research participants were included in this study.
Reporting on race, ethnicity, or other socially relevant groupings	No human research participants were included in this study.
Population characteristics	No human research participants were included in this study.
Recruitment	No human research participants were included in this study.
Ethics oversight	No human research participants were included in this study.

Note that full information on the approval of the study protocol must also be provided in the manuscript.

Field-specific reporting

Please select the one below that is the best fit for your research. If you are not sure, read the appropriate sections before making your selection.

[x] Life sciences [ ] Behavioural & social sciences [ ] Ecological, evolutionary & environmental sciences

For a reference copy of the document with all sections, see nature.com/documents/nr-reporting-summary-flat.pdf

Life sciences study design

All studies must disclose on these points even when the disclosure is negative.

Sample size	The sample size was determined based on the our prior experience and previous studies in the field as for in vivo experiments (Chen D., et al. Nat Commun. 12, 3644 (2021); Chu B., et al. Nat Cell Biol. 21, 579-591(2019); Yang X., et al. Cell Metab. 35, 1474-1490 (2023); Zhang Y., et al. Nat Cell Biol. 20, 1181-1192 (2018);Kim R., et al. 612, 338-346 (2022)). Unless otherwise stated, in vitro experiments were performed with three biological replicates, so n=3 was the number of sample size which is enough for in vitro experiments. Sample size of each experiment was provided in Figure legend.
Data exclusions	No data was excluded from this study.
Replication	All experiments were independently repeated three times with similar results
Randomization	6 weeks Nude mice were used for in vivo mouse models, and mice were randomly allocated into each experimental group. In vitro experiments were not randomized; all cell samples were analyzed equally without sub-sampling, so in in vitro experiments were no need for randomization.
Blinding	The tumor model development or tumor measurement was performed blinded by two individual investigators. Blinding was performed in the analysis of the IHC intensity score, which is quantified with automated quantitative software. For in vitro experiments, experiments design and analysis were conducted by two individual investigators, so two investigators were blinded on group allocation and data analysis, respectively.

Reporting for specific materials, systems and methods

We require information from authors about some types of materials, experimental systems and methods used in many studies. Here, indicate whether each material, system or method listed is relevant to your study. If you are not sure if a list item applies to your research, read the appropriate section before selecting a response.

Materials & experimental systems

n/a	Involved in the study
☐	☒ Antibodies
☐	☒ Eukaryotic cell lines
☒	☐ Palaeontology and archaeology
☐	☒ Animals and other organisms
☒	☐ Clinical data
☒	☐ Dual use research of concern
☒	☐ Plants

Methods

n/a	Involved in the study
☐	☒ ChIP-seq
☐	☒ Flow cytometry
☒	☐ MRI-based neuroimaging

Antibodies

Antibodies used

Following primary antibodies were used for co-IP assay and western blot analysis: Flag (Sigma-Aldrich, F3165, 1:5000 dilution; RRID:AB_259529); Vinculin (Sigma-Aldrich, V9131, 1:10000 dilution; RRID:AB_477629); Actin (Sigma-Aldrich, A3853, 1:5000 dilution; RRID:AB_262137); HA (Roche, 11867423001, 1:2000 dilution; RRID:AB_390918); NRF2 (Abcam, ab62352, 1:200 dilution; RRID:AB_944418); NRF2 (Cell signaling technology, 12721, 1:200 dilution; RRID:AB_2715528); SLC7A11(Cell signaling technology, 12691, 1:1000 dilution; RRID:AB_2687474); p53 (DO-1)(Santa Cruz Biotechnology, sc-126, 1:10000 dilution; RRID:AB_628082); GAS41(Santa Cruz Biotechnology, sc-393708, 1:200 dilution; RRID:AB_2892567); GCLC (Proteintech, 12601-1-AP, 1:10000 dilution; RRID:AB_2278734); NQO1(Proteintech, 11451-1-AP, 1:10000 dilution; RRID:AB_2298729); TIP60 (Proteintech, 10827-1-AP, 1:500 dilution; RRID:AB_2128431); FSP1(AMID) (Santa Cruz Biotechnology, sc-377120, 1:1000 dilution; RRID:AB_2893240); GPX4 (Abcam, ab125066, 1:1000 dilution; RRID:AB_10973901); DHODH (Proteintech, 14877-1-AP, 1;10000 dilution; RRID:AB_2091723).

Following primary antibodies were used for CUT&RUN and ChIP-qPCR analysis: CBP (Santa Cruz Biotechnology, sc-369; RRID:AB_631006), NRF2 (Abcam, ab62352; RRID:AB_944418); HA (Roche, 11867423001; RRID:AB_390918); Flag (Sigma-Aldrich, F3165; RRID:AB_259529); GAS41(Santa Cruz Biotechnology, sc-393708; RRID:AB_2892567).

Following primary antibody was used for IHC analysis: 4-Hydroxynonenal (4-HNE) (Abcam, ab46545, 1:200 dilution; RRID: AB_722490).

Following second antibodies were used for western blot analysis: Peroxidase AffiniPure"" Goat Anti-Mouse IgG (H-FL) (Jackson Immunoresearch, Cat#115-035-146, 1:5000 dilution; RRID:AB_2307392), Peroxidase AffiniPure"" Goat Anti-Rabbit IgG (H+L) (Jackson Immunoresearch, Cat#111-035-045, 1:5000 dilution; RRID:AB_2337938), and Goat Anti-Rat IgG(H+L) (SouthernBiotech, Cat#3050-05, 1:5000 dilution; RRID:AB_2795830).

Validation

All antibodies used in this study are commercially available and have been validated by manufacturer. All validation statements are available on the manufacturer's website. The RRID# and manufacturer' website of each antibody is provided below:
Flag (Sigma-Aldrich, F3165, RRID:AB_259529), https://www.sigmaaldrich.com/US/en/product/sigma/f3165
Vinculin (Sigma-Aldrich, V9131, RRID:AB_477629), https://www.sigmaaldrich.com/US/en/product/sigma/v9131
Actin (Sigma-Aldrich, A3853, RRID:AB_262137), https://www.sigmaaldrich.com/US/en/product/sigma/a3853
HA (Roche, 11867423001, RRID:AB_390918), https://www.sigmaaldrich.com/US/en/product/roche/roahaha
NRF2 (Abcam, ab62352, RRID:AB_944418), https://www.abcam.com/products/primary-antibodies/nrf2-antibody-ep1808yab62352.html
GPX4 (Abcam, ab125066, RRID:AB_10973901), https://www.abcam.com/products/primary-antibodies/glutathione-peroxidase-4-antibody-epncir144-ab125066.html
4-Hydroxynonenal (4-HNE) (Abcam, ab46545, RRID: AB_722490), https://www.abcam.com/products/primary-antibodies/4-hydroxynonenal-antibody-ab46545.html
NRF2 (Cell signaling technology, 12721, RRID:AB_2715528), https://www.cellsignal.com/products/primary-antibodies/nrf2-d1z9c-xprabbit-mab/12721
SLC7A11(Cell signaling technology, 12691, RRID:AB_2687474),https://www.cellsignal.com/products/primary-antibodies/xet-sle7a11-d2m7a-rabbit-mab/12691
p53 (DO-1)(Santa Cruz Biotechnology, se-126, RRID:AB_628082), https://www.scbt.com/p/p53-antibody-do-1?requestFrom=search
GAS41(Santa Cruz Biotechnology, sc-393708, RRID:AB_2892567), https://www.sebt.com/p/gas41-antibody-c-10?requestFrom=search
FSP1(AMID) (Santa Cruz Biotechnology, se-377120, RRID:AB_2893240), https://www.scbt.com/p/amid-antibody-b-6?requestFrom=search
CBP (Santa Cruz Biotechnology, sc-369, RRID:AB_631006) had been discontinued, https://www.scbt.com/p/cbp-antibody-a-22?requestFrom=search and antibody datasheets was shown on https://datasheets.scbt.com/sc-369.pdf
GCLC (Proteintech, 12601-1-AP,RRID:AB_2278734), https://www.ptglab.com/products/GCLC-Antibody-12601-1-AP.htm
NQO1(Proteintech, 11451-1-AP, RRID:AB_2298729), https://www.ptglab.com/products/NC1O1-Antibody-11451-1-AP.htm
TIP60 (Proteintech, 10827-1-AP, RRID:AB_2128431), https://www.ptglab.com/products/KATS-Antibody-10827-1-AP.htm
DHODH (Proteintech, 14877-1-AP, RRID:AB_2091723), https://www.ptglab.com/products/DHODH-Antibody-14877-1-AP.htm
Peroxidase AffiniPure"" Goat Anti-Mouse IgG (H+L) (Jackson Immunoresearch, Cat#115-035-146, RRID:AB_2307392), https://

www.jacksonimmuno.com/catalog/products/115-035-146
Peroxidase AffiniPure'"' Goat Anti-Rabbit IgG (H+L) (Jackson Immunoresearch, Cat#111-035-045, RRID:AB_2337938), https://www.jacksonimmuno.com/catalog/products/111-035-045
Goat Anti-Rat IgG(H+L) (SouthernBiotech, Cat#3050-05, RRID:AB_2795830), https://www.southernbiotech.com/goat anti rat igg h l mouse-ads-hrp-3050-05

Eukaryotic cell lines

Policy information about cell lines and Sex and Gender in Research

Cell line source(s)

293T ATCC Cat# CRL-3216
H1299 ATCC Cat# CRL-5803
A549 ATCC Cat# CCL-185
A375 ATCC Cat# CRL-1619
H460 ATCC Cat# HTB-177

Authentication

All cell lines were not authenticated.

Mycoplasma contamination

The cell lines were tested negative for mycoplasma contamination

Commonly misidentified lines
(See ICLAC register)

No cell line used in the study was found in the databases of commonly misidentified cell lines that are maintained by ICLAC.

Animals and other research organisms

Policy information about studies involving animals; ARRIVE guidelines recommended for reporting animal research, and Sex and Gender in Research

Laboratory animals

Six-week-old Nu/Nu mice(Charles River, 088; RRID:IMSR_CRL:088) were purchased from Charles River Laboratories for xenograft experiments. All the mice were housed in a temperature-controlled room (65-75°F) with 40-60% humidity, with a light/dark cycle of 12h/12h.

Wild animals

No wild animals were used in this study.

Reporting on sex

Female nude mice were used for xenograft experiments. To reduce fighting behavior, we used female nude mice. This study was not focused on sex-dependent phenotypes or mechanisms.

Field-collected samples

No field-collected samples were used in this study.

Ethics oversight

This study is compliant with the relevant ethical regulations for animal experiments. All experimental protocols were approved by the Institutional Animal Care and Use Committee (IACUC) of Columbia University under the supervision of the Institute of Comparative Medicine (ICM).

Note that full information on the approval of the study protocol must also be provided in the manuscript.

Plants

Seed stocks

No plants were included in this study.

Novel plant genotypes

No plants were included in this study.

Authentication

No plants were included in this study.

ChIP-seq

Data deposition

☒ Confirm that both raw and final processed data have been deposited in a public database such as GEO.

☒ Confirm that you have deposited or provided access to graph files (e.g. BED files) for the called peaks.

Data access links
May remain private before publication.

GSE256462 (https://www.ncbi.nlm.nih.gov/geo/query/acc.cgi?acc=GSE256462

Files in database submission	Xin061_NC12_NRF2_cpm.bw; Xin062_sg11_NRF2_cpm.bw Xin061_NC12_NRF2_1.fastq.gz; Xin061_NC12_NRF2_2.fastq.gz ; Xin062_sg11_NRF2_1.fastq.gz; Xin062_sg11_NRF2_2.fastq.gz
Genome browser session (e.g. UCSC)	No longer applicable.

Methodology

Replicates	One replicate was presented in CUT&RUN study
Sequencing depth	Xin061_NC12_NRF2, Total reads:17123430, Mapped reads, 15570624, 75bp, pair-end.<br>Xin062_sg11_NRF2, Total reads:19692100, Mapped reads, 4985219, 75bp, pair-end.
Antibodies	NRF2 (Abcam, ab62352; RRID:AB_944418)
Peak calling parameters	SEACR software (version 1.3) was used for peak calling. Overlapped peaks were extracted using BEDTools (version 2.29.2).
Data quality	Raw reads were trimmed to remove sequencing adaptors and low-quality reads using Trim Galore (version 0.6.7) with default parameters.
Software	All software used for CUT&RUN analysis included Trim Galore (version 0.6.7), Bowtie2 (version 2.2.4), BEDTools (version 2.29.2), bedGraphToBigWig (version 359), and SEACR2 (version 1.3).

Flow Cytometry

Plots

Confirm that:

☒ The axis labels state the marker and fluorochrome used (e.g. CD4-FITC).

☒ The axis scales are clearly visible. Include numbers along axes only for bottom left plot of group (a 'group' is an analysis of identical markers).

☒ All plots are contour plots with outliers or pseudocolor plots.

☒ A numerical value for number of cells or percentage (with statistics) is provided.

Methodology

Sample preparation	For in vitro cultured cell staining, cells were pre-treated with indicated compounds for the indicated time before further treatment or directly treated with indicated compounds at the indicated concentrations for the indicated time. Cells were incubated with 2 M BODIPYrm 581/591C11dye (ThermoFisher Scientific, D3861) for 30min at 37°C. Then cells were harvested, washed by 1xPBS twice, and resuspended in 500 L PBS, followed through a 35 m cell strainer (Falcon, 352235) for flow cytometry analysis. Lipid peroxidation levels were measured with Attune NxT Acoustic Focusing Cytometer (ThermoFisher Scientific) through the BL1channel by analyzing 10,000 cells.<br>For Xenografts-derived cells, isolated tumor tissues were cut into small enough pieces and digested with collagenase type I (Thermo Fisher Scientific, 17100017) at 37°C incubator for 1h. The contents were passed a 35 m cell strainer, rinsed with 1xPBS twice, and resuspended with 1xPBS. Finally, cells were stained with 2 M BODIPrm 581/591C11dye for 25 min at 37°C in the dark. Then, the stained cells were rinsed by 1xPBS twice and resuspended in 1xPBS for flow cytometry analysis. Lipid peroxidation levels were measured with Attune NxT Acoustic Focusing Cytometer (ThermoFisher Scientific) through the BL1channel by analyzing 10,000 cells.
Instrument	Attune NxT Acoustic Focusing Cytometer (Thermo Fisher Scientific)
Software	Flow.lo v10
Cell population abundance	At least 10,000 cells were analyzed for each sample.
Gating strategy	live cells--single cells positive staining cells.

☒ Tick this box to confirm that a figure exemplifying the gating strategy is provided in the Supplementary Information.
